# Pollen Emissions of Subpollen Particles and Ice Nucleating Particles

**DOI:** 10.1021/acsearthspacechem.3c00014

**Published:** 2023-04-27

**Authors:** Brianna H. Matthews, Alyssa N. Alsante, Sarah D. Brooks

**Affiliations:** † Department of Atmospheric Sciences, Texas A&M University, College Station, Texas 77843, United States; ‡ Department of Oceanography, Texas A&M University, College Station, Texas 77843, United States

**Keywords:** pollen, subpollen particles, emission factors, ice nucleation, live oak, ryegrass, giant ragweed

## Abstract

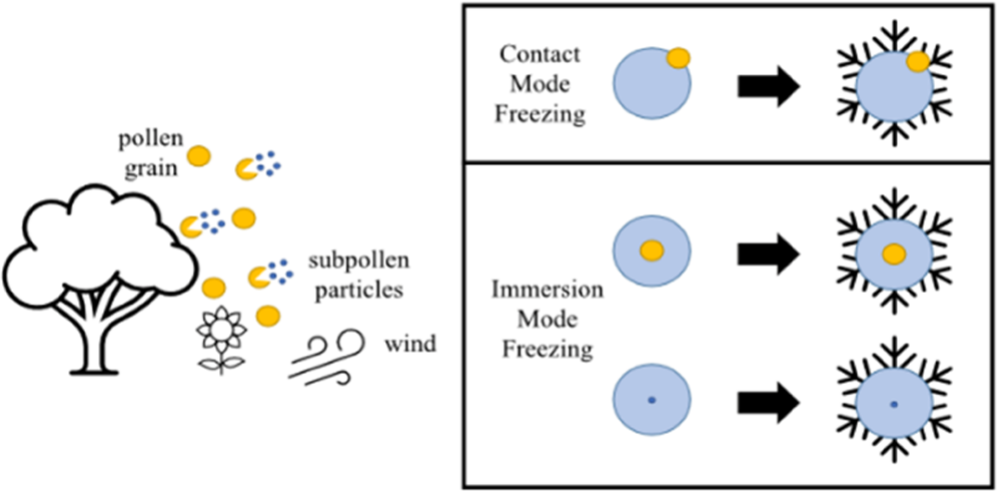

Pollen grains significantly contribute to the aerosol population, and levels are predicted to increase in the future. Under humid atmospheric conditions, pollen grains can rupture creating pollen grain fragments referred to as subpollen particles (SPPs) which are dispersed into the atmosphere with wind. In this laboratory study, SPP emission factors were determined for ryegrass, *Lolium* sp., and giant ragweed,*Ambrosia trifida*, in terms of the number of SPPs produced per pollen grain and the number of SPPs produced per m^2^, which were compared to previously measured live oak,*Quercus virginiana*, emission factors. The SPP emission factors were 4.9 × 10^13^ ± 4.3 × 10^13^ SPPs per m^2^ for ryegrass, 1.3 × 10^15^ ± 1.1 × 10^15^ SPPs per m^2^ for giant ragweed, and 1.1 × 10^15^ ± 1.6 × 10^15^ SPPs per m^2^ for live oak. SPPs and whole pollen grains from these species were evaluated for their ice nucleation efficiency in immersion and contact mode freezing. Measurements of the ice nucleation efficiency indicate that SPPs are weakly effective INPs in immersion mode, but that pollen grains represent a source of moderately efficient INPs in immersion and contact modes.

## Introduction

Projections for the end of the century show that pollen seasons could start up to 40 days earlier and last up to 19 days longer with temperature change, and the total annual emissions could increase 16–40% over the United States.^1^ Since pollen is currently a significant source to the aerosol population and will continue to be in the future, quantifying pollen emissions to the atmosphere is important for predicting the effects on cloud formation and climate as well as human health. Previous studies have demonstrated that pollen dispersal may be driven primarily by wind.^2,3^ When exposed to moisture, pollen grains become enlarged with water and osmotically rupture creating pollen grain fragments, referred to as subpollen particles (SPPs).^3−9^ Following pollen grain rupture, winds can disperse the SPPs into the atmosphere.^2,3,9^ Studies have shown that pollen grain rupture can produce high concentrations of SPPs, which can be lofted into the atmosphere.^3,10^

Given the magnitude of SPP emissions, accurate assessment of their potential to influence clouds by acting as cloud condensation nuclei (CCN) or ice nucleating particles (INPs) is important. It has been shown that SPPs activate as CCN at atmospherically relevant supersaturations comparable for background sulfate aerosol, and thus may contribute to the overall cloud droplet concentration.^11,12^ Experiments have also been performed to determine if SPPs can act as INPs, though additional details regarding the most effective physical mechanisms and representative temperatures of ice nucleation remain uncertain.^13−16^

While only 1 in 10^5^ or fewer particles in the atmosphere are able to act as INPs, it is important to identify significant sources that contribute aerosol to the atmosphere and evaluate these aerosol for their ability to contribute to the INP population.^17−20^ Ice nucleation can occur homogeneously through the freezing of pure water or solute droplets, or heterogeneously where ice formation is induced by the addition of a foreign surface called an INP.^21^ Homogeneous freezing of pure water occurs at temperatures colder than −38 °C.^19^ Heterogeneous ice nucleation can occur through different modes including deposition, contact, and immersion.^22,23^ Immersion mode freezing is generally considered the most dominant heterogeneous freezing path for ice nucleation in mixed-phase clouds.^24^ However, the onset of contact freezing has been shown to occur 4–5 °C warmer than immersion freezing for the same particle.^20,25−30^

A recent study found a correlation between increased ice nucleation efficiency and higher protein concentrations in SPPs.^14^ Cytoplasmic material composing the SPPs, such as starches and proteins,^6−9,11^ could enhance their ability to catalyze ice nucleation by acting as efficient INPs. Specific tree SPPs (including Manitoba maple, sycamore maple, gray alder, silver birch, red ash, red mulberry, Lombardy poplar, red oak, black oak, Arizona cypress, and common juniper) smaller than 450 nm have been observed to nucleate ice under relevant atmospheric conditions.^15^ Previous results showed that birch (*Betula alba*), oak (*Quercus rubra*), pine (*Pinus sylvestris*), and grass (*Dactylis glomerata*) pollen grains are able to activate as INPs in immersion mode freezing, and even more efficiently in contact mode freezing.^25^ It has been suggested that ice nucleation activity of pollen depends on the surface morphology and porosity of the pollen grain surface.^31^ Ice nucleation experiments analyzing 15 different species across four taxon showed that pollen grains with similar microtexture, resulting from close relatedness, can exhibit different freezing temperatures.^32^

In this study, SPP emission factors were determined for ryegrass, *Lolium* sp., and giant ragweed, *Ambrosia trifida*, using wind-driven experiments. Ryegrass was used in this study because it causes the most hypersensitivity among sweet grasses and is a large contributor to pollen in Texas.^33,34^ Similarly, giant ragweed was selected since it is well known as an allergen, and recent studies have found its pollen season has lengthened and the concentration of pollen has increased over the last several decades.^10,35−37^ The ryegrass and giant ragweed SPP emission factors were compared to live oak, *Quercus virginiana*, which we measured in a previous study.^3^ The ice nucleation efficiency of SPPs from all three species was investigated for immersion mode freezing. Additionally, the ice nucleation efficiency for pollen grains from each species was investigated for immersion mode and contact mode freezing. SPP emission factors and ice nucleating temperatures are valuable inputs to better represent pollen emissions in climate models and to elucidate the impacts on cloud formation, cloud lifetime, and precipitation.

## Experimental Methods

### Sample Collection

**Figure 1:**
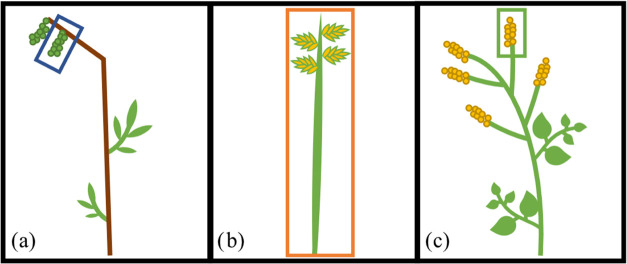
Different plant species used in experiments with the reproductive units shown. (a) A branch from a live oak tree. The blue box indicates a catkin. (b) A blade of ryegrass which is referred to as a spike. The orange box indicates the spike. (c) A branch from a giant ragweed plant. The green box indicates an inflorescence.

Similar to our previous work with live oak samples,^3^ ryegrass and giant ragweed samples were collected on or near the Texas A&M University campus in College Station, Texas. Figure 1 schematically illustrates these three plant species. Ryegrass samples were collected from May 19, 2021, to May 25, 2021. An area of grass containing many spikes was selected. The sample was dug up and placed into a glass container for transport back to the laboratory. Giant ragweed samples were collected from September 13, 2021, to October 5, 2021. After cutting, the branches were immediately placed into a bag and sealed. This reduced agitation to the branch prior to testing during transport to the laboratory by protecting the samples from direct wind. At the time of sample collection for each species, meteorological parameters including temperature, relative humidity, wind speed, wind direction, dew point temperature, and pressure were measured at the collection site using a Kestrel Weather Meter (Kestrel Model 4000), and values were cross-checked with data available from the nearby Easterwood airport (approximately 3.7 miles away). Additional notes were taken on precipitation conditions or cold/warm fronts that occurred prior to sample collection. Meteorological parameters measured at the time of sample collection were compared to the conditions used in the chamber during experiments. This data is provided in Supplementary Information Tables S1 and S2. In the laboratory, each sample was photographed. Figure 1 shows the plant anatomy for the three species included in this study. Since the three species are quite different, the reproductive units were counted for each sample before the experiment to serve as a normalizing factor. The number of catkins was counted for each live oak sample (Figure 1a), the number of spikes per area of ryegrass (Figure 1b), and the number of inflorescences for giant ragweed (Figure 1c). The counts of reproductive units were used in the data analysis to normalize the total SPP emissions by the number of reproductive units present in the sample.

Additional samples were collected and set aside to provide pollen grains for ice nucleation experiments included in this study. The samples were placed onto a table covered in aluminum foil that had been wiped with isopropyl alcohol. The samples were left on the table until pollen grains had deposited onto the foil. The pollen grains were collected and placed into 15 mL sealable centrifuge tubes and stored in a freezer at −20 °C until analyzed. This procedure was followed for live oak, ryegrass, and giant ragweed samples. Live oak pollen was collected during the previous study^3^ and therefore, additional pollen grains were not collected in the current study. As not enough ryegrass pollen was collected for analysis, ryegrass pollen purchased from Pharmallegra was used in ice nucleation experiments. The purchased ryegrass pollen was the same species of ryegrass used in the chamber experiments. Previous studies have also used purchased pollen for different experiments including ice nucleation.^14−16,32,38,39^ One caveat to this approach is that commercially available pollen may be slightly different than the fresh emissions from our chamber.^40^ However, this was the only option available to us at the time of the experiments.

### Pollen Chamber

There are limited studies that investigate the specific environmental conditions required for SPP release. During periods of high humidity or precipitation, pollen grains become enlarged with water and osmotically rupture producing SPPs. After rain events, the relative humidity will decrease, causing anthers to open and expose the ruptured pollen grain fragments.^9^ It has often been assumed that SPP emissions require a high relative humidity for osmostic pollen grain rupture to occur followed by a decrease in relative humidity and a wind disturbance leading to SPP dispersal. However, more recent results have shown that SPPs can be dispersed by wind even under constant humidity conditions.^3^ In this study, wind-driven SPP release was quantified for ryegrass and giant ragweed by conducting experiments at a constant relative humidity (70% or greater) and cycling fans on and off to simulate wind disturbances which occur in a natural setting.

The pollen chamber was designed and assembled in the laboratory at Texas A&M University and has been described in detail in previous work,^3^ therefore, it is briefly discussed here. The chamber walls were constructed using 2 mm thick PFA Teflon to reduce static which could cause particles to cling to the walls of the chamber. The clear acrylic chamber lid and base were also covered in PFA Teflon. Inside the chamber were four fans to simulate wind throughout the experiments. To mimic natural sunlight, four sets of LED light strips were facing the chamber. Additionally, the chamber was suspended from the ceiling and surrounded by a metal frame to prevent individuals from walking close to the chamber and introducing static. Similar chambers have been used as a proxy for outdoor conditions.^41−44^ Results showed that twigs cut from a donor tree and placed into the chamber were similar in flowering and pollen production to the donor trees.^41^

A sample branch was placed onto the chamber base between the three small fans for each experiment. The sample was secured to the chamber using industrial Velcro to ensure the sample was stationary and did not contact the lid or walls of the chamber. After each experiment the chamber base was detached, and the sample was removed. The base of the chamber was cleaned with isopropyl alcohol to remove pollen which had collected during the experiment to prevent previous pollen from contributing to subsequent experiments. Weekly the entire chamber was cleaned by wiping the inside surfaces with isopropyl alcohol.

As with previous chamber experiments,^3^ when dry air was introduced into the chamber, the relative humidity of the chamber never reached the reduced relative humidity of the incoming air. This was attributed to the sample releasing moisture in the chamber through transpiration. Giant ragweed branches were placed into a bottle of water which was placed into the chamber for each experiment. The volume of water in the bottle was measured before the experiment began and after the experiment to determine the amount of water lost through transpiration. On average, over a single giant ragweed experiment the consumption of water was 103.0–121.3 mL. Since transpiration rates are affected by light level, temperature, soil water, wind, and humidity,^45^ changing the conditions within the chamber would cause the rate of transpiration to increase. Experiments included in this study held light level, temperature, and humidity constant during an experiment which helped minimize transpiration variability between experiments.

In our previous study,^3^ experiments were conducted to determine the aerosol contribution from a live oak branch and leaves in the absence of pollen. These experiments provided an estimate of the sources of aerosol other than SPPs. It was determined that the total aerosol contributions from the branch and leaves was on average 29.5% of the total aerosol measured in an experiment containing a branch, leaves, and catkins.^3^ Since the branches used in these experiments were collected after the pollen season had ended, there were more leaves present than the branches that were used in SPP experiments. Therefore, the aerosol emissions measured from the branch and the leaves would be a higher estimate than the aerosol contributed from the branches and leaves during the SPP experiments. The average total contribution from the branch alone was 25.3%. These results highlight that the aerosol measured in each experiment mainly originated directly from the pollen grain and can be considered SPPs. Consequently, all aerosol measured during experiments were assumed to be SPPs.

### Instrument Measurements

Experimental setup was described previously^3^ and is briefly discussed here. A steady flow of zero-grade air was supplied to the pollen chamber from a cylinder of compressed air. To create and control humidified air, zero-grade air was passed through a Perma Pure humidifying tube (Perma Pure LLC Model MH-110-24F-4) before entering the chamber. The relative humidity was measured prior to entering the chamber as well as inside of the chamber using relative humidity probes (Rotronic Model HC2A-S). An Acu-rite weather station sensor (Acu-Rite Weather Station model 02099) was placed into the chamber to confirm the relative humidity of the probe inside the chamber was correct. Upon exiting the chamber, the flow was dried using a desiccant tube and then directed to the instruments. The instruments included a condensation particle counter (CPC, TSI, Inc., Model 3786 or CPC, TSI, Inc., Model 3750), a portable aerosol spectrometer (PAS, GRIMM Model 1.108), and a PIXE cascade impactor (PIXE International Corporation Model I-1L). Filters from the PIXE cascade impactor were used for offline ice nucleation analysis reported in this study. The CPC measured the concentration of total aerosol (approximately 0.01–1 μm diameter) exiting the chamber. The maximum concentration of SPPs released was determined by plotting the CPC concentration as a time series. A total concentration was calculated by summing the concentration over the entire 18 h experiment. The PAS measured size bins from 0.3 to 20 μm with 5 of the bins measuring submicron (0.3–1.0 μm) sizes.

### Quantifying Emissions from Wind-Driven SPP Release

Prior to each experiment, the chamber was purged to remove background particles which had entered while the sample was placed inside the chamber. The purge cycle lasted for 36 min and had an incoming flowrate of dry air at 2.0 × 10^4^ cm^3^ per minute (CCM). This duration was selected to remove at least 95% of the background particles according to the exponential decay equation. Following the purge cycle, the chamber was set to either a high relative humidity (>95%) or a lower relative humidity (67–80%) for 3 h without fans and then 3 h with fans. These conditions would repeat for three cycles. The fans were powered using a power supply and operated at 12 V, which was their maximum rating to provide a wind speed inside the chamber of 1.8 m s^−1^. The environmental relative humidity at the time of sample collection ranged from 25.0 to 92.0% with an average of 61.5%. The environmental wind speed during sample collection ranged from 0.4 to 6.7 m s^–1^ with an average of 3.4 m s^–1^. The chamber relative humidity was similar to the environmental relative humidity. The wind speed inside the chamber was also comparable to the ambient environment but at the lower end of the environmental measurements.

### Ice Nucleation Experiments

Ice nucleation measurements were conducted at Texas A&M University as described in detail in previous work.^26,46^ In this study, the ice nucleation efficiency of SPPs was investigated for immersion mode freezing and the ice nucleation efficiency of pollen grains was investigated for immersion and contact mode freezing. SPPs and pollen grains were assigned the following terms based on their mean ice nucleation temperature: −32 to −25 °C were described as “weakly efficient,” −25 to −20 °C as “moderately efficient,” −20 to −10 °C were described as “efficient” and >−10 °C as “highly efficient.” Since the size cut for the PIXE cascade impactor stage was selected to sample particles ranging from 60 nm to 1.0 μm, it was assumed this only sampled SPPs. To confirm the collection of SPPs, PIXE cascade impactor samples were imaged by scanning electron microscope (SEM) as shown in Supplemental Figure S3. While the nominal size cutoff used for the PIXE cascade impactor was 60 nm to 1 μm, a few larger particles were also collected. Nevertheless, the impaction surface was densely populated by particles in the submicron range, which was attributed to SPPs. The images confirm that SPPs were collected onto the impactor stages during chamber experiments (Supplemental Figure S3). To transfer the SPPs collected during chamber experiments to a slide for ice nucleation, a 2 μL droplet of ultrahigh-performance liquid chromatography (UHPLC) grade water (Sigma-Aldrich) was added to the aerosol sample collected on the aluminum foil disk from the PIXE cascade impactor (PIXE, Inc.). The droplet was transferred from the aluminum foil disk onto a slide with a hydrophobic coating. The slide was placed in a cold stage (Linkam Scientific Instruments Model LTS 120) mounted onto an optical microscope (Olympus Model BX43F). The cold stage temperature was controlled using a T96 controller and a water circulation pump (Linkam Scientific Instruments) using LabSpec 6.2 software (HORIBA Instruments, Inc.). To help maintain the droplet volume, a constant flow of humidified nitrogen, created by passing dry nitrogen gas through a bubbler containing high-performance liquid chromatography (HPLC) water, was mixed with dry nitrogen in a glass mixing chamber. Nitrogen gas flow rates were controlled using mass flow controllers (Alicat Scientific Model MC-10 SLPM-D(N2)) and the humidity was monitored using a dew point hygrometer (EdgeTech Model Dew Prime II). The droplet was cooled at a rate of 1 °C/min until the temperature reached −40 °C. After −40 °C was reached, the droplet was warmed to 5 °C where the temperature was held constant for 1 min to allow complete thawing. The standard experimental procedure is to maintain the droplet was maintained for 25 freezing and thawing cycles. In this study, the number of complete cycles varied from 19 to 25 for all samples included in the analysis. The droplet became opaque when completely frozen making it possible to visualize and record the freezing temperature using images that were taken every 6 seconds (Syncerity CCD, HORIBA Instruments, Inc.).

In addition to the analysis of SPPs, whole pollen grains were also characterized. In immersion mode ice nucleation experiments, pollen grains were spread onto a slide with a hydrophobic coating. Using the microscope to view the pollen grains, a syringe was used to clear off excess pollen grains until the area had only one pollen grain present. A 2 μL UHPLC water droplet was then placed onto the pollen grain, and the slide was then placed inside the stage. The steps mentioned previously for SPP immersion ice nucleation experiments were followed. To determine contact mode freezing, a 2 μL UHPLC water droplet was placed onto a hydrophobic slide. A syringe was used to transfer pollen grains from the sealable centrifuge tube to the surface of the water droplet by lightly touching the syringe tip to the surface of the droplet. Using the microscope, it was confirmed that pollen grains had been transferred to the droplet. The slide was then placed into the stage and the freeze/thaw cycles mentioned previously were performed. UHPLC water process blanks were also used in ice nucleation experiments for comparison. For a blank test, UHPLC water was transferred from the stock solution into a 15 mL sealable centrifuge tube and then a 2 μL droplet was placed on the stage for an ice nucleation measurement as above.

### Data Analysis

#### SPP Emission Factors—Ryegrass

SPP emission factors were calculated for each ryegrass experiment as the number of SPPs per pollen grain and the number of SPPs per m^2^. To determine the number of SPPs per pollen grain, eq 1 was applied to the CPC data collected from the entire duration of the experiment. The number of SPPs per pollen grain was calculated from the chamber data and a manual count of the spikes per sample. These values were then used to find the number of SPPs per pollen grain. The number of pollen grains per spike was determined by Prieto-Baena et al.^47^ to be 5,271,037. This number was calculated by analyzing 30 plants and averaging the number of pollen grains per spike. It was assumed that 70% of the pollen grains in an experiment would rupture. This factor is based on the results from previous work in which 70% of pollen grains exposed to moisture were observed to rupture within 5 min.^9,10,48^ In eq 1, Conc is the CPC concentration, *V* is the chamber volume, *T* is the number of turnovers, and *PG* is the number of pollen grains. The number of turnovers is defined as the number of times the total air volume within the chamber has been completely replaced.
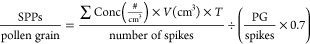
1Next, the SPP emission factor per m^2^ was calculated using eq 2. An area of ryegrass was collected and used in the equation for the area of the sample. In eq 2, Conc is the CPC concentration, *V* is the chamber volume, and *T* is the number of turnovers.
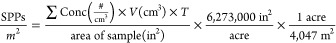
2The calculations in this study include several assumptions which could impact the SPP emission factors. First, the chamber experiments were conducted under a constant simulated wind speed without any wind gusts. Consequently, the observed SPP emission factors are most representative of emissions during similar wind conditions in nature. Second, the plant density was found using visual analysis of the local area in the vicinity of the Texas A&M campus which could also vary with region and land use. Despite these experimental caveats, the SPP emission factors in this study are the most representative to date.

Additionally, hourly SPP emission rates were determined. The number of SPPs was summed over a burst, a burst is defined as the period of time that SPP concentration was at least 250% higher than the background concentration, and divided over the 3 h period resulting in the number of SPPs per hour. This is considered the 1 h SPP peak emission rate. The hourly mean SPP emission rate was also determined by taking the total SPPs counted over an experiment and dividing by the 18 h duration of an experiment.

#### SPP Emission Factors—Giant Ragweed

**Figure 2:**
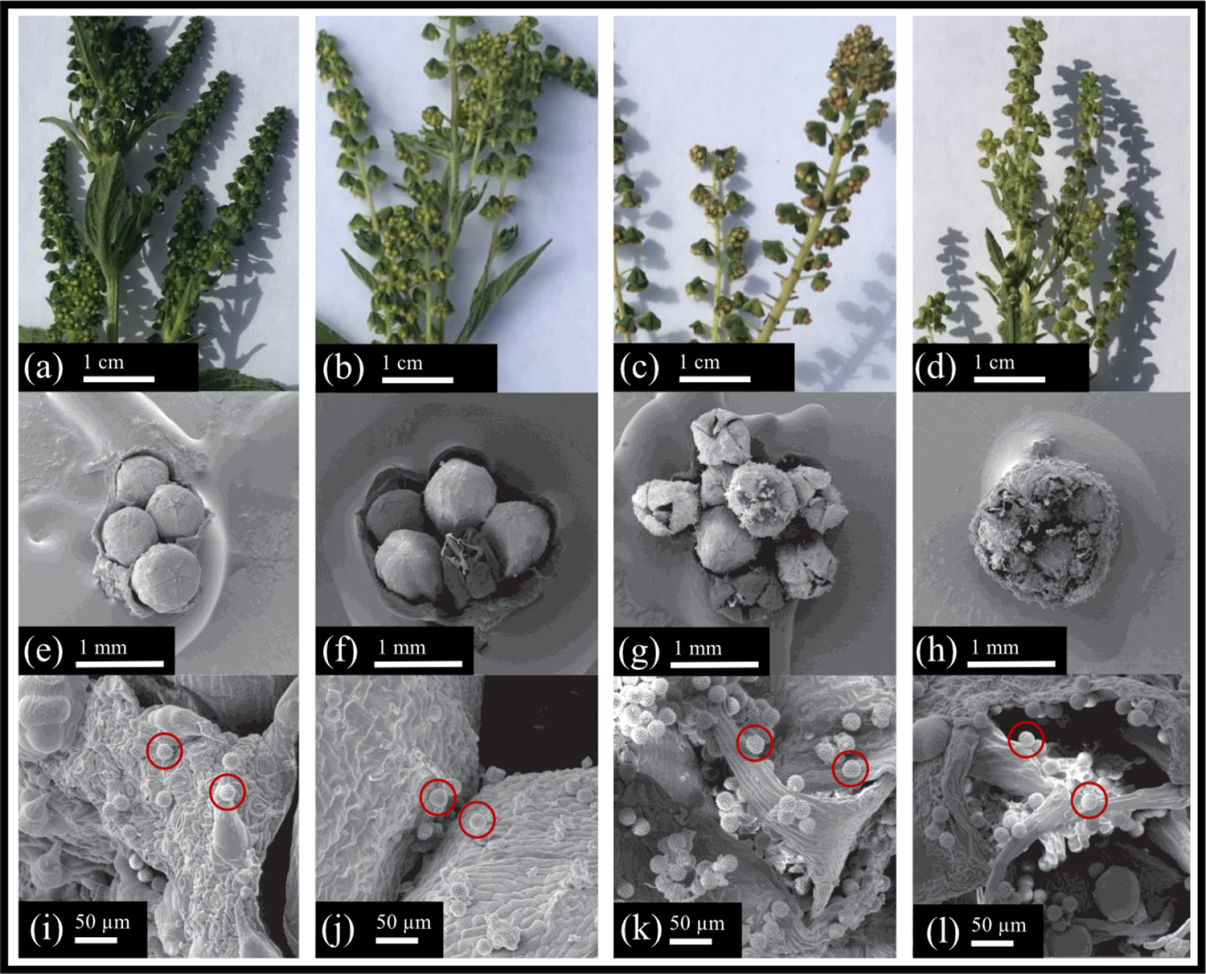
Scanning electron microscope (SEM) images of ragweed samples. (a–d) Ragweed samples as seen by the human eye. (e–h) SEM images of a bract from each ragweed sample with the flowers. (i–l) SEM images of the pollen grains located within the bracts. The first column shows a young giant ragweed sample, the second column shows an adolescent giant ragweed sample, the third column shows a mature giant ragweed sample, and the fourth column shows the images from a common ragweed sample. In (i–l), the red circles indicate individual pollen grains. Giant ragweed samples were used in chamber experiments.

Similarly, SPP emission factors were calculated for each giant ragweed experiment as the number of SPPs per pollen grain and the number of SPPs per m^2^. To determine the number of SPPs per pollen grain, eq 3 was applied to the CPC data collected from the entire duration of the experiment. The number of SPPs per pollen grain was calculated from the chamber data and a manual count of the inflorescences per sample. These values were then used to find the number of SPPs per pollen grain. Figure 2 shows samples of common ragweed and giant ragweed that were photographed using a SEM. Figure 2a–d shows the inflorescences on sample branches. Inflorescences are covered with many bracts which are shown in Figure 2e–h. The bracts contain the flowers which house the anthers. The number of anthers per inflorescence was determined using SEM photographs in previous work on common ragweed from Bankowski and Katz.^49^ SEM photographs from this study were used to determine the mean flower size for common ragweed was 0.42 mm and the mean flower size for giant ragweed was 0.65 mm. The pollen grains are located on anthers within the flowers. While flower and anther size vary between common and giant ragweed, the pollen grain size for each type of ragweed is similar. The number of anthers per inflorescence of 40 determined for common ragweed by Bankowski and Katz^49^ was used in the calculations. The literature value for pollen grains per anther for common ragweed was used to calculate the pollen grains per anther for giant ragweed. The range of values for pollen grains per anther of 3408 ± 2127^50^ for common ragweed was scaled by 1.55, ratio of 0.65:0.42 mm, to account for additional pollen grains on a larger giant ragweed anther. To provide a range of pollen grains per anther, the standard deviation was subtracted and added to the mean to give a lower and upper value. This range of values for pollen grains per anther was used in eq 3 to determine a range of values for the SPPs per pollen grain. In eq 3, Conc is the CPC concentration, *V* is the chamber volume, *T* is the number of turnovers, and *PG* is the number of pollen grains.
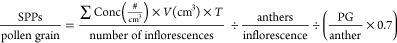
3Next, the SPP emission factor per plant was calculated using eq 4. The number of branches sampled and the total number of branches were counted during collection. The number of plants per area was determined by counting the number of giant ragweed plants in a specified area. Using these values, the SPPs per plant were determined for each sample. In eq 4, Conc is the CPC concentration, *V* is the chamber volume, and *T* is the number of turnovers. The number of branches collected refers to the number of branches that were cut to be placed into the chamber for an experiment, which is different than the total number of branches on the plant. Both numbers are needed to calculate the SPPs per plant emission factor.
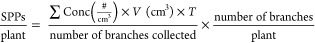
4Lastly, the SPPs per m^2^ emission factors were calculated using eq 5. The SPPs per plant determined in eq 4 were used along with conversion factors to convert to units of m^2^.
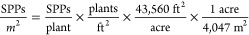
5The hourly mean SPP emission rates and 1 h SPP peak emission rates were also determined for the giant ragweed experiments.

#### Ice Nucleation

Our experimental process includes cycling the samples through a series of freeze-thaw cycles in order to obtain up to 25 independent determinations of the ice nucleation temperature for each sample (Supplemental Table S4). The fraction of the total droplets frozen versus the total number of droplets at a given temperature is referred to as fraction frozen. Fraction frozen was calculated using eq 6,^51^ where *P*(*T*) is the probability of freezing, *N*_f_ is the number of frozen droplets, and *N*_0_ is the total number of droplets.
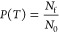
6Equation 7 shows the calculation of the number of ice nucleation active sites per pollen grain.^51^
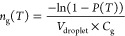
7In this study, the volume of the droplet, *V*_droplet_, is 2 μL and *C*_g_ is the count of pollen grains used in each individual experimental setup where each experiment was repeated three times. While multiple replicates were performed for each type of grain and mechanism (Supplemental Table S4), for each individual experimental setup, a single grain is used; thus, *C*_g_ is 1.

Next, values of *n*_g_(*T*) were converted to number of ice nucleation active sites per reproductive unit, *n*_r_(*T*), using eq 8.
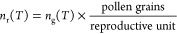
8Additionally, values of *n*_r_(*T*) were converted to number of ice nucleation active sites per m^2^, *n*_a_(*T*), using eq 9. The reproductive units per plant and the number of plants per m^2^ was determined differently depending upon each species. For live oak, the number of reproductive units per tree and number of trees per m^2^ was used. The number of reproductive units per area (similar to eq 2) for ryegrass was used in the calculations. Lastly, the number of reproductive units per giant ragweed branch collected was used in the calculations to get the total per plant and then extrapolate out to m^2^ (similar to eqs 4 and 5).
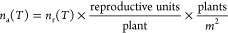
9Equations 1–9 were used to obtain the results presented in the section below.

## Results and Discussion

**Figure 3:**
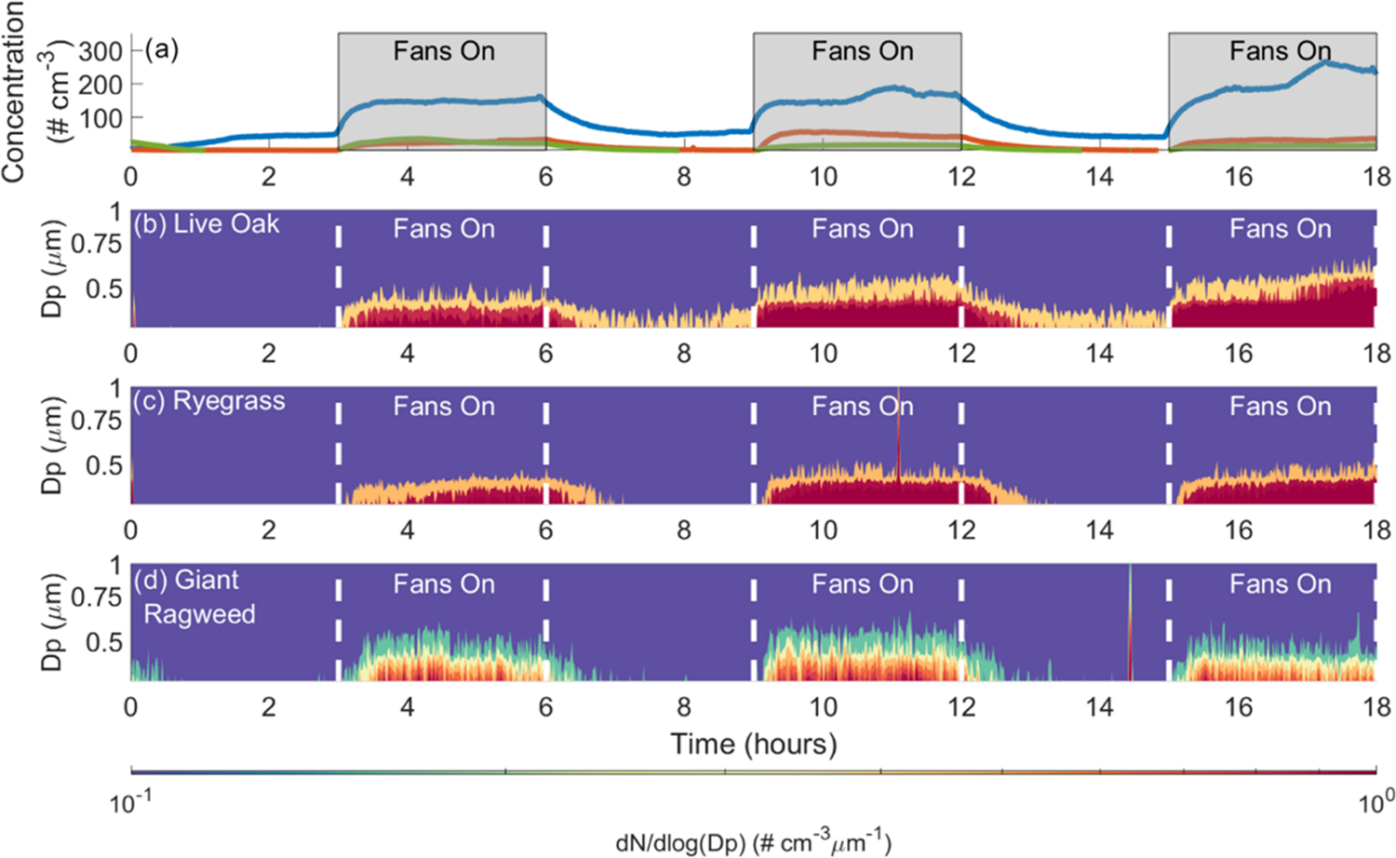
Representative measurements of wind-driven SPP release at high relative humidity (>95%). (a) Aerosol concentration (# cm^–3^) as measured by the CPC is shown for a representative live oak, ryegrass, and giant ragweed experiment in blue, orange, and green, lines, respectively. Concentrations were averaged over 1 min. Times when the fans were in operation are shaded in gray. (b–d) Normalized aerosol size distributions measured by the portable aerosol spectrometer (PAS). Size bins measured particles ranging from 0.35 to 1.0 μm. Concentrations were averaged over 1 min. Times when the fans were in operation are indicated by white dashed lines. Data are shown for a representative live oak, ryegrass, and giant ragweed experiment in (b)–(d), respectively.

To quantify wind-driven SPP emissions, 15 independent 18 h chamber experiments were conducted as summarized in Supplemental Table S5. Of these experiments, 12 were conducted at a higher relative humidity (>95%) and three were conducted at a lower relative humidity (67–80%). Figure 3 shows data collected from a representative experiment from each of the three species quantifying wind-driven SPP release at a high relative humidity (>95%). In Figure 3a, aerosol measured by the CPC during live oak (blue line), ryegrass (orange line), and giant ragweed (green line) experiments are shown. The live oak data was reported in our previous study.^3^ The gray boxes indicate where the fans were operated. In all experiments, a burst of SPPs was observed each time the fans were turned on resulting in an increased aerosol concentration. Once the fans were stopped, the concentrations decreased to less than approximately 50, 20, and 10 per cm^3^ for live oak, ryegrass, and giant ragweed, respectively. Therefore, a burst was defined as the 3 h period where fans were turned on inside the chamber. Given the pollen grain size for live oak, ryegrass, and giant ragweed are 28, 25, and 17 μm, we assume all particles counted by the CPC in the size range of 0.010–1.0 μm are SPPs. As can be seen, live oak SPP concentrations were higher than the concentrations observed in the ryegrass and giant ragweed experiments.

Figure 3b–d shows the normalized particle concentration size distribution data collected by the PAS during the live oak (Figure 3b), ryegrass (Figure 3c), and giant ragweed (Figure 3d) experiments. The PAS size distributions were normalized to account for the width of each bin size and reported as a normalized concentration distribution. The data showed the majority of particles measured were submicron (<1 μm diameter) and few supermicron particles were counted. Even though the PAS measures particles up to 20 μm in diameter, the data shown in this figure only includes particles less than 1 μm in diameter which accounted for >98% of the particles measured by the PAS. These results were consistent with our previous measurements of SPP emissions from live oak,^3^ as well as previous studies that showed the majority of giant ragweed SPPs were submicron.^10^

Particles less than 4 μm in size are able to penetrate the lower respiratory system.^52^ Therefore, our studies imply that SPPs can become lodged in the lower respiratory tract causing more health effects than pollen grains.^5,6,8,10,53^ Fine particles cause more pulmonary inflammation and are retained longer in the lung.^54^ Larger particles such as pollen grains, which are 20–40 μm in diameter, would deposit in the nasal cavity. While both pollen grains and SPPs have health effects, SPPs are more detrimental to human health due to their size and large concentration.

**Figure 4:**
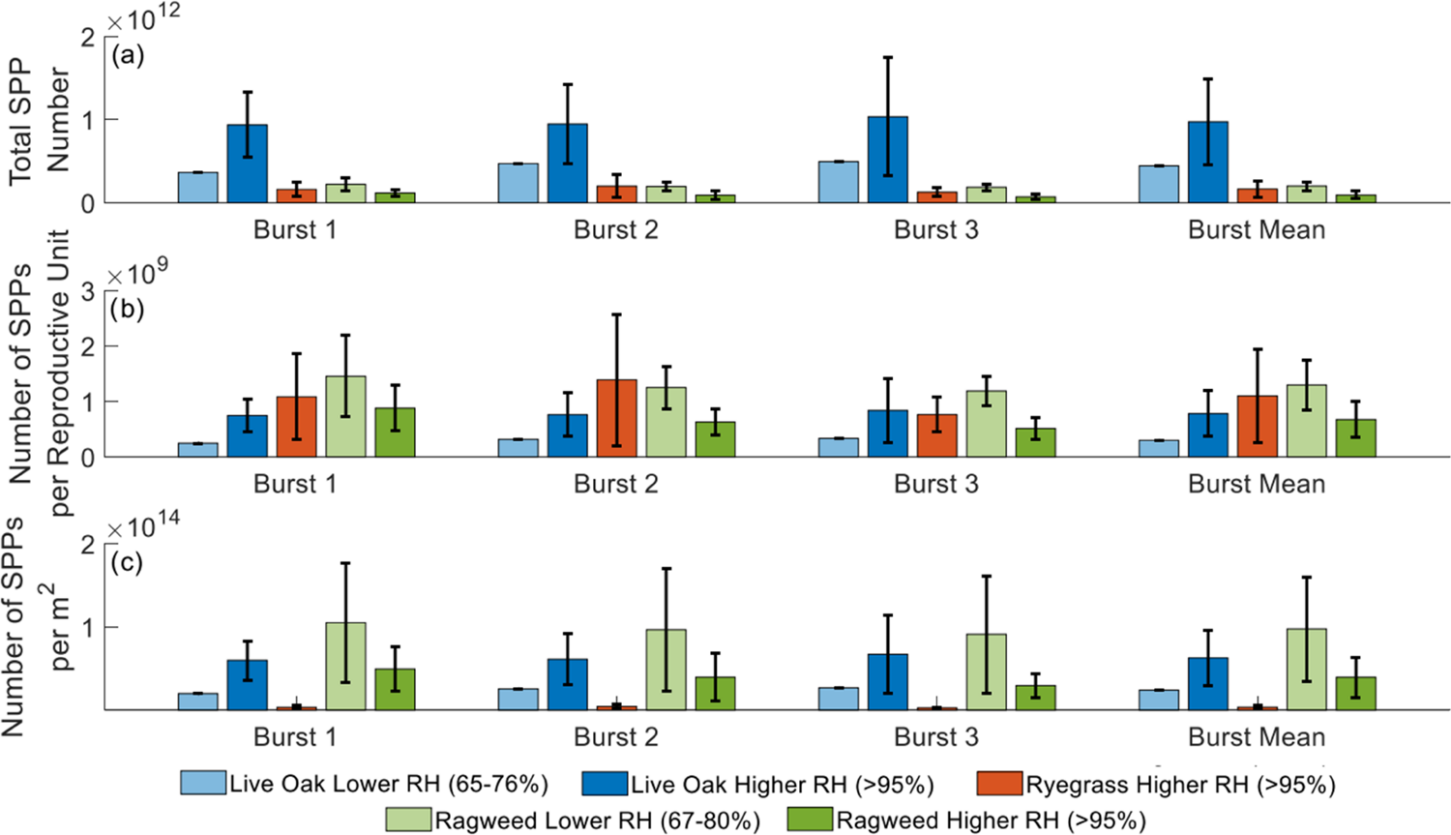
SPP number concentrations measured by the CPC. (a) Total SPP number per burst and the mean SPP number across the bursts for each species and relative humidity are shown. The species include live oak, ryegrass, and giant ragweed indicated by the blue, orange, and green bars, respectively. The darker-colored bars represent the mean over the high humidity (>95%) wind-driven SPP release experiments, and the lighter-colored bars represent the mean over the lower humidity (65–76% for live oak and 67–80% for giant ragweed) wind-driven SPP release experiments. (b) Number of SPPs per reproductive unit in a burst and the mean SPPs per reproductive unit for each species and relative humidity are shown. (c) Number of SPPs per m^2^ and the mean SPPs per m^2^ for each species and relative humidity are shown.

Total SPP emissions observed during each burst for each of the three species are shown in Figure 4a. The darker-colored bars show data at a high relative humidity (>95%) and the lighter-colored bars show data at a lower relative humidity (65–76% for live oak and 67–80% for giant ragweed). Total SPP emissions were much higher in the live oak experiments than in the experiments involving other species (Figure 4a). However, Figure 4b illustrates the fact that the normalized live oak emissions per reproductive unit are similar to emissions from ryegrass and giant ragweed because the number of catkins on each live oak branch was more numerous than the number of reproductive units for the other two species. Lastly, Figure 4c shows the SPP emission factors in concentration per m^2^ for each species. In this panel, the emission factors from live oak and giant ragweed are significantly higher than ryegrass in bursts 1 and 2 using analysis of variance (ANOVA) and the 95% confidence level. However, ryegrass is not statistically different in burst 3. Live oak and ragweed are not statistically different in any of the three bursts. The highest total SPP emissions are produced from live oak, followed by giant ragweed, and then ryegrass at a high relative humidity. However, at a lower relative humidity, the highest total SPP emissions are produced from giant ragweed and then live oak. No experiments were conducted for ryegrass at a lower relative humidity (<80%) since ryegrass pollen season had ended and all samples collected had been used in experiments. For live oak experiments, there was not a significant difference between the total SPP emissions observed under the high vs low ranges of relative humidity. For giant ragweed experiments, there was a statistically significant difference between the total SPP emissions at a high relative humidity compared to a lower relative humidity at a 95% confidence level. To summarize, Table 1 shows the mean emission factors by species for SPPs per pollen grain and SPPs per m^2^. There is a difference between the SPP emission factors determined for each species. However, the differences between SPP emission factors in terms of SPPs per pollen grain were not explained by the size of the pollen grain. Giant ragweed pollen grains have the smallest diameter of the species included in this study but had the largest SPP per pollen grain emission factor. Supplemental Table S5 shows the emission factors for each individual experiment as well as the hourly mean SPP emission rates.

**Table 1:** Mean SPP Emission Factors

species	SPPs/pollen grain	SPPs/m^2^
live oak, RH > 95%	7.7 × 10^4^ ± 7.2 × 10^4^	1.6 × 10^15^ ± 2.0 × 10^15^
live oak, RH 65–76%	3.1 × 10^4^	6.3 × 10^14^
ryegrass, RH > 95%	5.5 × 10^3^ ± 2.0 × 10^3^	4.9 × 10^13^ ± 4.3 × 10^13^
giant ragweed, RH > 95%	1.7 × 10^5^ ± 1.3 × 10^5^	9.2 × 10^14^ ± 5.0 × 10^14^
giant ragweed, RH 67–80%	3.4 × 10^5^ ± 2.6 × 10^5^	2.3 × 10^15^ ± 1.7 × 10^15^

The SPP per pollen grain emission factors from this study (calculated using eqs 1 and 3) can be compared to previous SPP emission factors. In the current study, the mean number of SPPs produced per pollen grain for giant ragweed was 2.2 × 10^5^. A previous study determined that 1,400 SPPs per pollen grain were produced for giant ragweed,^10^ which is 2 orders of magnitude smaller than the emission factors determined in this study. Additionally, in the current study, the mean number of SPPs produced per pollen grain for ryegrass was 5.5 × 10^3^. Experiments by Suphioglu et al.^6^ found that 700 SPPs per pollen grain were produced from ryegrass samples which is 1 order of magnitude lower than those determined in the current study. This shows that previous studies may have undercounted the number of SPPs produced per pollen grain. However, these previous studies did use a different method for generating SPPs. Stone et al.^10^ used conventional SPP release in a chamber study where 500 μL of deionized water was sprayed onto the pollen grains concurrent with shaking the stems to act as wind in 5 min intervals followed by relative humidity cycling. Suphioglu et al.^6^ placed fresh ryegrass pollen into distilled water and shook the pollen grains to induce osmotic shock which ruptured the pollen grains and produced SPPs. The ruptured pollen grains were filtered, and the number of starch granules released was counted. It was assumed that each starch granule produced a single SPP. The method used in our study for counting SPPs generated directly from plants in our pollen chamber is more representative of pollen grain rupture and SPP dispersal in an ambient environment than these previous estimates.

A recent modeling study estimated how four types of vegetation including ragweed modify clouds and precipitation.^48^ Assuming 10^3^ SPPs were produced per pollen grain, no significant change in precipitation was observed. However, assuming 10^6^ SPPs per pollen grain were present and served as CCN, the CCN concentration increases 5–20% over a pollen-free scenario causing a reduction in precipitation of 32% on average in the continental United States. However, the SPP emission factors used in that study were not based on experimental methods. The SPP emission factors reported here are within the limits used in the modeling study but given the broad range between the upper and lower limits, changes to precipitation cannot be inferred at this time. Emission factors reported here provide more refined values for future modeling studies.

**Figure 5:**
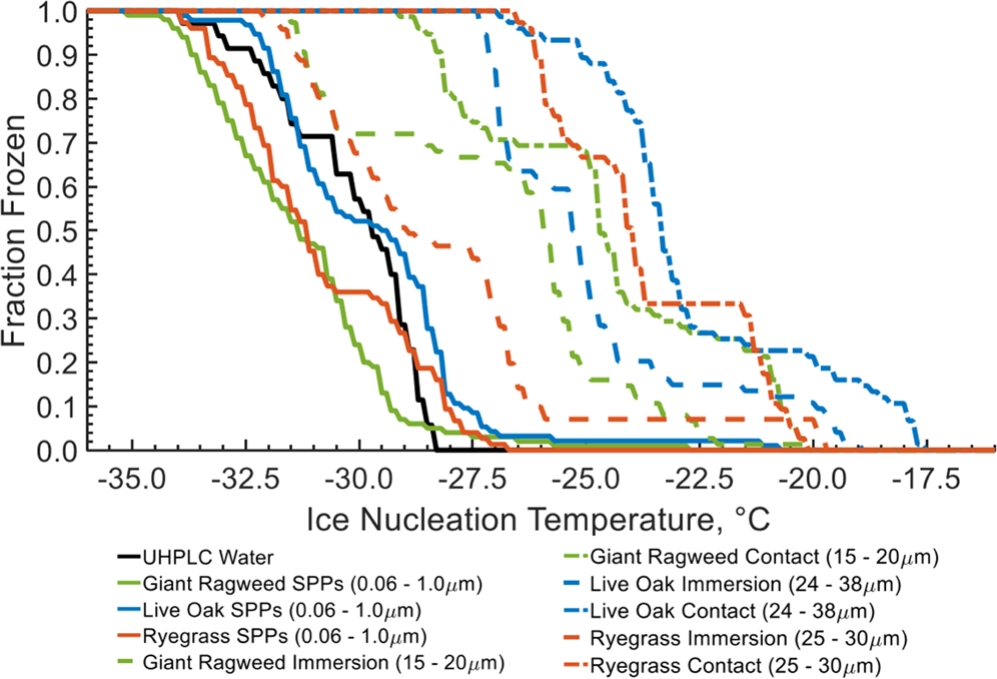
Fraction frozen at each temperature collected during multiple ice nucleation experiments for each species and freezing mode. The black line shows the fraction frozen for a pure UHPLC water process blank. The blue, orange, and green lines show the fraction frozen for live oak, ryegrass, and giant ragweed experiments, respectively. The solid line shows fraction frozen of SPPs, the dashed line shows fraction frozen for whole pollen grains in immersion mode, and the dashed dotted line shows fraction frozen for whole pollen grains in contact mode.

The fraction of droplets frozen as a function of temperature is shown in Figure 5 which includes the data points from all experiments. From these data, there is a clear divide between the freezing temperatures of SPPs and pollen grains. Pollen grains facilitate freezing at warmer temperatures with a mean temperature of −26.7 °C for immersion mode ice nucleation experiments and a mean temperature of −23.7 °C for contact mode ice nucleation experiments. Between species, there is variation in freezing temperature. Live oak pollen grains had mean freezing temperatures of −25.0 and −22.7 °C for immersion mode and contact mode experiments. Ryegrass had mean freezing temperatures of −28.3 and −23.7 °C for immersion mode and contact mode experiments. Lastly, giant ragweed had mean freezing temperatures of −27.0 and −24.6 °C for immersion mode and contact mode. In contrast, the droplets containing SPPs froze at −29.8, −30.8, and −31.3 °C for live oak, ryegrass, and giant ragweed, respectively. By comparison, process blanks froze at a mean temperature of −30.7 °C. This indicates that pollen grains are more efficient than SPPs as INPs.

**Figure 6:**
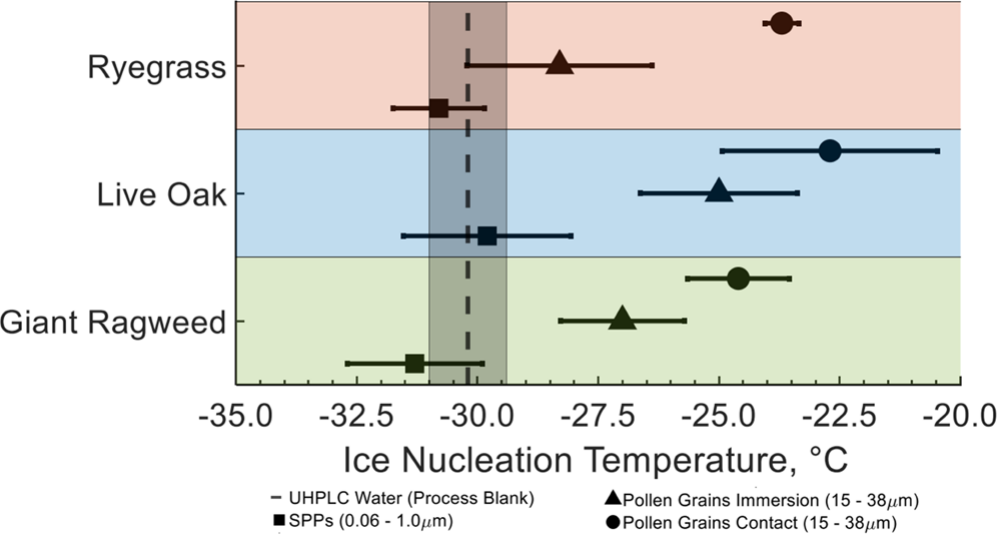
Experimental means and pooled standard deviations of freezing temperature. The orange-shaded area indicates ryegrass experiments, the blue-shaded area indicates live oak experiments, and the green-shaded area indicates giant ragweed experiments. The square marker shows the mean and pooled standard deviation for freezing experiments of SPPs, the triangle marker shows the mean and pooled standard deviation for whole pollen grains in immersion mode freezing experiments, and the circle marker shows the mean and pooled standard deviation for whole pollen grains in contact mode freezing experiments. The black dashed line shows the freezing temperature for a pure UHPLC water process blank, and the shading around it indicates the pooled standard deviation.

In Figure 6, the mean and pooled standard deviation of ice nucleation temperature are summarized. These values are also included in Table 2. In this figure, there is also a clear difference in the ice nucleation temperature between SPPs and pollen grains. Among the pollen grain experiments, there is also a shift in the ice nucleation temperature between pollen grains in contact mode freezing and immersion mode freezing. For each of the three species in this study, there is a statistical difference, defined using ANOVA and the 95% confidence level, between the ice nucleation temperature of the SPPs, pollen grains in contact mode, and pollen grains in immersion mode. This indicates that the ice nucleation temperature measured for each freezing mechanism was different. Additionally, the statistical significance was determined for SPPs of all three species, pollen grains in contact mode for all three species, and pollen grains in immersion mode for all three species. Live oak SPP ice nucleation temperatures were statistically different than giant ragweed and ryegrass ice nucleation temperatures. There was no statistical difference between the SPP ice nucleation temperatures for giant ragweed and ryegrass. Similarly, for contact mode freezing temperatures live oak was statistically different but ryegrass and giant ragweed were not statistically different. For immersion mode freezing temperatures, all three species were statistically significant. From these data, the live oak samples were always statistically different from the other species, but all of the species were only statistically different for immersion mode freezing of pollen grains. Supplementary Table S4 includes the mean ice nucleation temperatures, number of replicates, and data points for each sample type used in ice nucleation experiments. Additionally, live oak and ryegrass SPP ice nucleation temperatures were not statistically different than the UHPLC water process blank. However, giant ragweed SPP ice nucleation temperatures were statistically different than the process blanks with colder freezing temperatures. These results indicate that within the limitations of our experiments, no evidence that SPPs facilitate ice nucleation was observed.

**Table 2:** Mean Ice Nucleation Temperatures for SPPs and Pollen Grains

	freezing temperature (°C)		
species	SPPs—immersion	pollen grains—immersion	pollen grains—contact
live oak	–29.8 ± 1.7	–25.0 ± 1.6	–22.7 ± 2.2
ryegrass	–30.8 ± 1.0	–28.3 ± 1.9	–23.7 ± 0.4
giant ragweed	–31.3 ± 1.4	–27.0 ± 1.3	–24.6 ± 1.1

These results determined that SPPs were weakly effective INPs which contradicts the results from previous studies. However, previous studies used filtrate instead of SPPs for experiments. One study extracted birch (*Betula pendula*) filtrate and then tested the filtrate for ice nucleation in immersion mode after being washed.^14^ The number of washes ranged from one to seventy and as the number of washes increased the ice nucleation temperature decreased. After just one wash, the ice nucleation temperature was approximately −20 °C compared to a mean temperature of −30.7 °C in our study. Another study also tested filtrate in immersion mode from nine different deciduous tree species included manitoba maple (*Acer negundo*), sycamore maple (*Acer pseudoplatanus*), gray alder (*Alnus incana*), silver birch (*Betula pendula*), red ash (*Fraxinus pennsylvanica*), red mulberry (*Morus rubra*), lombardy poplar (*Populus nigra v. italica*), red oak (*Quercus rubra*), black oak (*Quercus velutina*), Arizona cypress (*Cupressus arizonica*), and common juniper (*Juniperus communis*).^15^ The study also found the ice nucleation temperatures of filtrate to be warmer, with all species completely freezing by −20 °C, than the ice nucleation temperatures measured in our current study. However, these previous studies extracted filtrate by adding whole pollen grains to water and then shaking the solution until the pollen grains ruptured then filtering the sample to remove large fragments or unruptured pollen grains. By comparison, SPPs collected on PIXE impactor stages during our pollen experiments represent only the naturally aerosolized materials and may lack some components of the whole pollen grains. The method generating filtrate or the different species being studied could contribute to the warmer ice nucleation temperatures determined in previous studies compared to the current study.

Additionally, a previous study determined ice nucleation temperatures for pollen grains in both contact mode and immersion mode experiments. That study included pollen grains from birch (*Betula alba*), oak (*Quercus rubra*), pine (*Pinus sylvestris*), and one grass pollen (*Dactylis glomerata*).^25^ The median ice nucleation temperatures for immersion mode experiments were −13.8, −15.8, and −16.2 °C for birch, oak, and grass respectively. While the median ice nucleation temperatures for contact mode experiments were −11.9, −13.4, and −13.6 °C for birch, oak, and grass respectively. In the current study, the median ice nucleation temperatures for immersion mode experiments were −25.3, −29.0, and −26.0 °C for live oak, ryegrass, and giant ragweed. Additionally, in the current study, the median ice nucleation temperatures for contact mode experiments were −23.4, −24.0, and −24.8 °C for live oak, ryegrass, and giant ragweed. However, there were differences in the pollen species, sample processing, and ice nucleation experiment setup between the previous study and the current study. The previous study did not include any of the same species included in this study and the samples were standardized dried pollen. The Diehl et al.^25^ study also used a vertical wind tunnel with freely suspended supercooled droplets for ice nucleation experiments. These differences could explain why the ice nucleation temperatures are colder in the current study than in the previous study.

**Figure 7:**
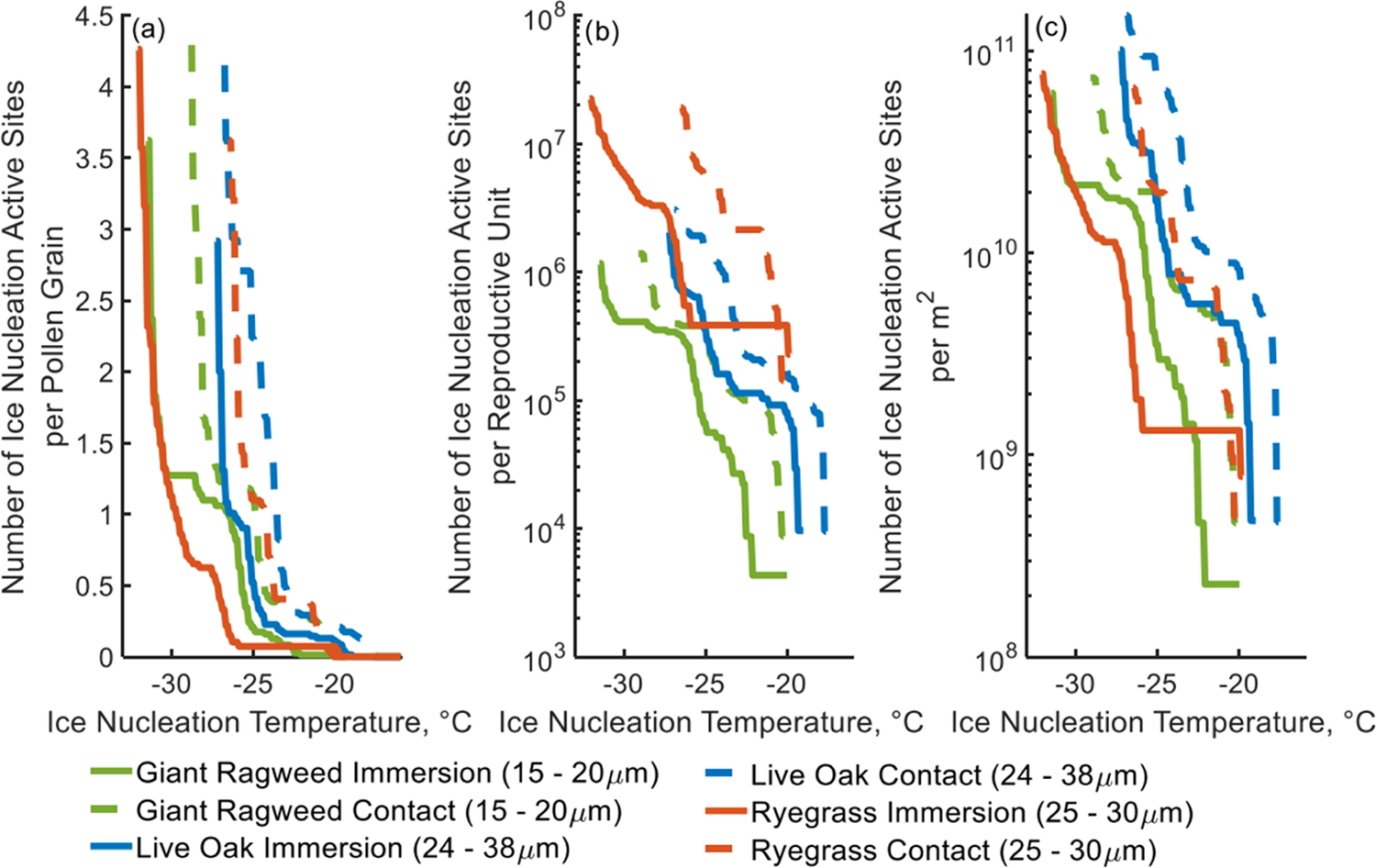
Number of ice nucleation active sites. (a) Number of ice nucleation active sites per pollen grain. Blue lines indicate live oak pollen grains, orange lines indicate ryegrass pollen grains, and green lines indicate giant ragweed pollen grains. The solid lines show the number of ice nucleation active sites from immersion mode freezing, and the dashed lines show the number of ice nucleation active sites from contact mode freezing. (b) Number of ice nucleation active sites per reproductive unit. (c) Number of ice nucleation active sites per m^2^.

Next, we calculated the overall concentrations of INP produced by each species involved in this study. Figure 7 shows the number of ice nucleation active sites per pollen grain (Figure 7a), per reproductive unit (Figure 7b), and per m^2^ (Figure 7c) which were calculated using eqs 7–9. In Figure 7a, the number of ice nucleation active sites per pollen grain does not seem large with mean values of 0.29, 0.25, and 0.22 for giant ragweed, live oak, and ryegrass respectively, but when extrapolated over an entire square meter the mean number of ice nucleation active sites is significant. In the general aerosol population, only 1 in 10^5^ or fewer particles are able to act as INPs in the atmosphere.^17−20^ Adding the mean number of ice nucleation active sites per square meter from pollen grains of 4.9 × 10^9^, 8.8 × 10^9^, and 4.0 × 10^9^ per m^2^ for giant ragweed, live oak, and ryegrass, respectively (shown in Figure 7c), would be a significant contribution to the total available ice nucleation sites provided by the total aerosol population.

## Conclusions

In this study, pollen grain rupture and wind-driven SPP dispersal were simulated in a series of laboratory studies. The observed SPP emission factors were 4.9 × 10^13^ ± 4.3 × 10^13^ SPPs per m^2^ for ryegrass, 1.3 × 10^15^ ± 1.1 × 10^15^ SPPs per m^2^ for giant ragweed, and 1.1 × 10^15^ ± 1.6 × 10^15^ SPPs per m^2^ for live oak. In this chamber study, the concentration of SPPs produced per pollen grain was up to 2 orders of magnitude higher than previously reported emission factors. These results suggest previous studies may have undercounted total SPP emissions for atmospherically relevant conditions. Variations between the species and conditions included in this study highlight the importance of characterizing additional species under a wider range of conditions to more accurately quantify SPP emission factors.

Ryegrass, giant ragweed, and live oak impact human health as allergens. The pollen grain size for live oak, ryegrass, and giant ragweed are 28, 25, and 17 μm, and cannot travel beyond the human upper respiratory tract. The vast majority of SPPS observed in this study were in the submicron diameter size range. Consequently, they have the potential to deposit in the lower respiratory system, be retained for substantial lengths of time, and cause more pulmonary inflammation.

SPPs and pollen grains were examined for their impact on ice cloud formation by determining the number of ice nucleation active sites for all three species. When the number of ice nucleation active sites was extrapolated out over a square meter, it was shown that pollen grains could contribute significantly to the INP population. Ice nucleation results indicated that pollen grains are more efficient than SPPs as INPs. From this study, the mean ice nucleation temperature for pollen grains in contact mode was −22.7, −23.7, and −24.6 °C for live oak, ryegrass, and giant ragweed, respectively. The mean ice nucleation temperature for pollen grains in immersion mode was −25.0, −28.3, and −27.0 °C for live oak, ryegrass, and giant ragweed, respectively. Lastly, the mean ice nucleation temperature for SPPs was −29.8, −30.8, and −31.3 °C for live oak, ryegrass, and giant ragweed, respectively. When comparing the ice nucleation temperatures for SPPs to the UHPLC process water blanks, live oak and ryegrass SPP ice nucleation temperatures were not statistically different than the UHPLC water process blanks. However, giant ragweed SPP ice nucleation temperatures were statistically different than the process blanks with lower freezing temperatures. Therefore, SPPs have either little or no ability to initiate ice nucleation. Variations between the live oak, ryegrass, and giant ragweed samples analyzed emphasize the importance of more broadly characterizing the ice nucleating properties of pollen grains and SPPs.

The ice nucleation temperatures determined in this study can be used in modeling to better represent the contributions from SPPs and pollen grains to the INP population. Since the ice nucleation temperatures between species and ice nucleation mechanisms (contact mode and immersion mode) were statistically different, one data set cannot be applied across species and freezing mechanisms to accurately represent pollen grains and SPPs. Laboratory measurements need to be performed to characterize the ice nucleation of different species and different ice nucleation freezing mechanisms. Results from this study indicate that SPPs have a greater impact on human health and the CCN population while pollen grains have a more important role in ice cloud formation. This study provides realistic estimates of the concentrations and ice nucleating temperatures of pollen grains and SPPs which have previously been unavailable. Based on our results, pollen represents a significant and increasing source of biogenic ice nucleating particles.

## Supporting Information Available

Meteorological parameters measured during ryegrass and ragweed sample collection (Tables S1 and S2); SEM photograph of a PIXE cascade impactor filter (Figure S3); data from ice nucleation experiments (Table S4); and ryegrass and giant ragweed wind-driven SPP emission factors and rates (Table S5) (PDF)

## References

[ref1] Zhang Y.; Steiner A. L. Projected climate-driven changes in pollen emission season length and magnitude over the continental United States. Nat. Commun. 2022, 13, 1234 10.1038/s41467-022-28764-0. 35292649 PMC8924258

[ref2] Visez N.; Chassard G.; Azarkan N.; Naas O.; Senechal H.; Sutra J. P.; Poncet P.; Choel M. Wind-induced mechanical rupture of birch pollen: Potential implications for allergen dispersal. J. Aerosol. Sci. 2015, 89, 77–84. 10.1016/j.jaerosci.2015.07.005.

[ref3] Hendrickson B. N.; Alsante A. N.; Brooks S. D. Live oak pollen as a source of atmospheric particles. Aerobiologia 2022, 39, 51–67. 10.1007/s10453-022-09773-4.

[ref4] Eisikowitch D.; Woodell S. R. Some aspects of pollination ecology of Armeria maritima (Mill.) Willd. in Britain. New Phytol. 1975, 74, 307–322. 10.1111/j.1469-8137.1975.tb02619.x.

[ref5] Schäppi G. F.; Suphioglu C.; Taylor P. E.; Knox R. B. Concentrations of the major birch tree allergen Bet v 1 in pollen and respirable fine particles in the atmosphere. J. Allergy Clin. Immunol. 1997, 100, 656–661. 10.1016/S0091-6749(97)70170-2. 9389296

[ref6] Suphioglu C.; Singh M. B.; Taylor P.; Bellomo R.; Holmes P.; Puy R.; Knox R. B. Mechanism of grass-pollen-induced asthma. Lancet 1992, 339, 569–572. 10.1016/0140-6736(92)90864-y. 1347092

[ref7] Taylor P. E.; Flagan R. C.; Miguel A. G.; Valenta R.; Glovsky M. M. Birch pollen rupture and the release of aerosols of respirable allergens. Clin. Exp. Allergy 2004, 34, 1591–1596. 10.1111/j.1365-2222.2004.02078.x. 15479275

[ref8] Grote M.; Valenta R.; Reichelt R. Abortive pollen germination: a mechanism of allergen release in birch, alder, and hazel revealed by immunogold electron microscopy. J. Allergy Clin. Immunol. 2003, 111, 1017–1023. 10.1067/mai.2003.1452. 12743566

[ref9] Taylor P. E.; Flagan R. C.; Valenta R.; Glovsky M. M. Release of allergens as respirable aerosols: A link between grass pollen and asthma. J. Allergy Clin. Immunol. 2002, 109, 51–56. 10.1067/mai.2002.120759. 11799365

[ref10] Stone E. A.; Mampage C. B. A.; Hughes D. D.; Jones L. M. Airborne sub-pollen particles from rupturing giant ragweed pollen. Aerobiologia 2021, 37, 625 10.1007/s10453-021-09702-x.

[ref11] Steiner A. L.; Brooks S. D.; Deng C. H.; Thornton D. C. O.; Pendleton M. W.; Bryant V. Pollen as atmospheric cloud condensation nuclei. Geophys. Res. Lett. 2015, 42, 3596–3602. 10.1002/2015gl064060.

[ref12] Mikhailov E. F.; Ivanova O. A.; Nebosko E. Y.; Vlasenko S. S.; Ryshkevich T. I. Subpollen Particles as Atmospheric Cloud Condensation Nuclei. Izv. Atmos. Ocean. Phys. 2019, 55, 357–364. 10.1134/s000143381904008x.

[ref13] Burkart J.; Gratzl J.; Seifried T. M.; Bieber P.; Grothe H. Isolation of subpollen particles (SPPs) of birch: SPPs are potential carriers of ice nucleating macromolecules. Biogeosciences 2021, 18, 5751–5765. 10.5194/bg-18-5751-2021.

[ref14] Burkart J.; Gratzl J.; Seifried T. M.; Bieber P.; Grothe H. Subpollen particles (SPP) of birch as carriers of ice nucleating macromolecules. Biogeosci. Discuss 2021, 2021, 1–15. 10.5194/bg-2021-8.

[ref15] Gute E.; Abbatt J. P. D. Ice nucleating behavior of different tree pollen in the immersion mode. Atmos. Environ. 2020, 231, 117488 10.1016/j.atmosenv.2020.117488.

[ref16] Gute E.; David R. O.; Kanji Z. A.; Abbatt J. P. D. Ice Nucleation Ability of Tree Pollen Altered by Atmospheric Processing. ACS Earth Space Chem. 2020, 4, 2312–2319. 10.1021/acsearthspacechem.0c00218.

[ref17] DeMott P. J.; Prenni A. J.; Liu X.; Kreidenweis S. M.; Petters M. D.; Twohy C. H.; Richardson M.; Eidhammer T.; Rogers D. Predicting global atmospheric ice nuclei distributions and their impacts on climate. Proc. Natl. Acad. Sci. 2010, 107, 11217–11222. 10.1073/pnas.0910818107. 20534566 PMC2895116

[ref18] Rogers D. C.; DeMott P. J.; Kreidenweis S. M.; Chen Y. Measurements of ice nucleating aerosols during SUCCESS. Geophys. Res. Lett. 1998, 25, 1383–1386. 10.1029/97GL03478.

[ref19] Kanji Z. A.; Ladino L. A.; Wex H.; Boose Y.; Burkert-Kohn M.; Cziczo D. J.; Krämer M. Overview of ice nucleating particles. Meteorol. Monogr. 2017, 58, 1.1–1.33. 10.1175/AMSMONOGRAPHS-D-16-0006.1.

[ref20] Vali G. Ice nucleation—A review. In Nucleation and atmospheric aerosols 1996; Elsevier, 1996; pp 271–279.

[ref21] Pruppacher H. R.; Klett J. D.; Wang P. K. Microphysics of Clouds and Precipitation; Taylor & Francis, 1998.

[ref22] Hoose C.; Mohler O. Heterogeneous ice nucleation on atmospheric aerosols: a review of results from laboratory experiments. Atmos. Chem. Phys. 2012, 12, 9817–9854. 10.5194/acp-12-9817-2012.

[ref23] Vali G. Nucleation Terminology. Bull. Amer. Meteorol. Soc. 1985, 66, 1426–1427.

[ref24] Tobo Y. An improved approach for measuring immersion freezing in large droplets over a wide temperature range. Sci. Rep. 2016, 6, 32930 10.1038/srep32930. 27596247 PMC5011777

[ref25] Diehl K.; Matthias-Maser S.; Jaenicke R.; Mitra S. K. The ice nucleating ability of pollen: Part II. Laboratory studies in immersion and contact freezing modes. Atmos. Res. 2002, 61, 125–133. 10.1016/s0169-8095(01)00132-6.

[ref26] Fornea A. P.; Brooks S. D.; Dooley J. B.; Saha A. Heterogeneous freezing of ice on atmospheric aerosols containing ash, soot, and soil. J. Geophys. Res.-Atmos. 2009, 114, 12 10.1029/2009jd011958.

[ref27] Shaw R. A.; Durant A. J.; Mi Y. Heterogeneous surface crystallization observed in undercooled water. J. Phys. Chem. B 2005, 109, 9865–9868. 10.1021/jp0506336. 16852192

[ref28] Durant A. J.; Shaw R. A. Evaporation freezing by contact nucleation inside-out Geophys. Res. Lett. 2005, 32 (20), 10.1029/2005GL024175.

[ref29] Pitter R. L.; Pruppacher H. A wind tunnel investigation of freezing of small water drops falling at terminal velocity in air. Q. J. R. Meteorol. Soc. 1973, 99, 540–550. 10.1002/qj.49709942111.

[ref30] Pruppacher H.; Klett J. Microstructure of Atmospheric Clouds and Precipitation. In Microphysics of Clouds and Precipitation; Springer, 2010; pp 10–73.

[ref31] Diehl K.; Quick C.; Matthias-Maser S.; Mitra S. K.; Jaenicke R. The ice nucleating ability of pollen - Part I: Laboratory studies in deposition and condensation freezing modes. Atmos. Res. 2001, 58, 75–87. 10.1016/s0169-8095(01)00091-6.

[ref32] Pummer B. G.; Bauer H.; Bernardi J.; Bleicher S.; Grothe H. Suspendable macromolecules are responsible for ice nucleation activity of birch and conifer pollen. Atmos. Chem. Phys. 2012, 12, 2541–2550. 10.5194/acp-12-2541-2012.

[ref33] Vusthepalli P. S. G.; Vusthepalli G.; Manne A.; et al. Comprehensive Study on Key Pollen Allergens. J. Pure Appl. Microbiol. 2022, 16, 110–115. 10.22207/JPAM.16.1.26.

[ref34] Evers G. W. Introduction to Annual Ryegrass; Crop Science Society of America, 1995.

[ref35] Ziska L.; Knowlton K.; Rogers C.; Dalan D.; Tierney N.; Elder M. A.; Filley W.; Shropshire J.; Ford L. B.; Hedberg C.; et al. Recent warming by latitude associated with increased length of ragweed pollen season in central North America. Proc. Natl. Acad. Sci. 2011, 108, 4248–4251. 10.1073/pnas.1014107108. 21368130 PMC3053965

[ref36] Ziska L. H.; Makra L.; Harry S. K.; Bruffaerts N.; Hendrickx M.; Coates F.; Saarto A.; Thibaudon M.; Oliver G.; Damialis A.; et al. Temperature-related changes in airborne allergenic pollen abundance and seasonality across the northern hemisphere: a retrospective data analysis. Lancet Planet. Health 2019, 3, e124–e131. 10.1016/S2542-5196(19)30015-4. 30904111

[ref37] Damialis A.; Traidl-Hoffmann C.; Treudler R. Climate Change and Pollen Allergies. Biodiversity and Health in the Face of Climate Change, 2019; pp 47–66.

[ref38] Gute E.; Abbatt J. P. Oxidative processing lowers the ice nucleation activity of Birch and Alder pollen. Geophys. Res. Lett. 2018, 45, 1647–1653. 10.1002/2017GL076357.

[ref39] Murray K. A.; Kinney N. L.; Griffiths C. A.; Hasan M.; Gibson M. I.; Whale T. F. Pollen derived macromolecules serve as a new class of ice-nucleating cryoprotectants. Sci. Rep. 2022, 12, 12295 10.1038/s41598-022-15545-4. 35854036 PMC9296471

[ref40] Swanson B.; Freeman M.; Rezgui S.; Huffman J. A. Pollen classification using a single particle fluorescence spectroscopy technique. Aerosol Sci. Technol. 2022, 57, 112–133. 10.1080/02786826.2022.2142510.

[ref41] Jung S.; Zhao F.; Menzel A. Establishing the twig method for investigations on pollen characteristics of allergenic tree species. Int. J. Biometeorol. 2021, 65, 1983–1993. 10.1007/s00484-021-02154-5. 34043087 PMC8536639

[ref42] Polgar C.; Gallinat A.; Primack R. B. Drivers of leaf-out phenology and their implications for species invasions: insights from T horeau’s C oncord. New Phytol. 2014, 202, 106–115. 10.1111/nph.12647. 24372373

[ref43] Sønsteby A.; Heide O. M. Chilling requirements of contrasting black currant (Ribes nigrum L.) cultivars and the induction of secondary bud dormancy. Sci. Hortic. 2014, 179, 256–265. 10.1016/j.scienta.2014.09.038.

[ref44] Vitasse Y.; Basler D. Is the use of cuttings a good proxy to explore phenological responses of temperate forests in warming and photoperiod experiments?. Tree Physiol. 2014, 34, 174–183. 10.1093/treephys/tpt116. 24488858

[ref45] Holding D. R.; Streich A. M. Plant Growth Processes: Transpiration, Photosynthesis, and Respiration, University of Nebraska Cooperative Extension, 2013.

[ref46] Wilbourn E. K.; Thornton D. C. O.; Ott C.; Graff J.; Quinn P. K.; Bates T. S.; Betha R.; Russell L. M.; Behrenfeld M. J.; Brooks S. D. Ice Nucleation by Marine Aerosols Over the North Atlantic Ocean in Late Spring. J. Geophys. Res.-Atmos. 2020, 125, 17 10.1029/2019jd030913.

[ref47] Prieto-Baena J. C.; Hidalgo P. J.; Domínguez E.; Galán C. Pollen production in the Poaceae family. Grana 2003, 42, 153–159. 10.1080/00173130310011810.

[ref48] Wozniak M. C.; Solmon F.; Steiner A. L. Pollen Rupture and Its Impact on Precipitation in Clean Continental Conditions. Geophys. Res. Lett. 2018, 45, 7156–7164. 10.1029/2018gl077692.

[ref49] Bankowski V.; Katz D. Estimates of common ragweed pollen production for urban ragweed plants. Retrieved from The University of Michigan Undergraduate Research Journal 2018, 12, 27–32.

[ref50] Šaulienė I.; Veriankaitė L.; Šaulys A. Biometrical assessment of ragweed (Ambrosia artemisiifolia L.). Žemdirbystė 2012, 99, 319–326.

[ref51] Vali G.; DeMott P.; Möhler O.; Whale T. A proposal for ice nucleation terminology. Atmos. Chem. Phys. 2015, 15, 10263–10270. 10.5194/acp-15-10263-2015.

[ref52] Phalen R. F.; Phalen R. N. Introduction to Air Pollution Science. In A Public Health Perspective; Jones & Bartlett Learning: Burlington, Mass, 2013.

[ref53] Pazmandi K.; Kumar B. V.; Szabo K.; Boldogh I.; Szoor A.; Vereb G.; Veres A.; Lanyi A.; Rajnavolgyi E.; Bacsi A. Ragweed subpollen particles of respirable size activate human dendritic cells. PLoS One 2012, 7, e52085 10.1371/journal.pone.0052085. 23251688 PMC3522620

[ref54] Schraufnagel D. E. The health effects of ultrafine particles. Exp. Mol. Med. 2020, 52, 311–317. 10.1038/s12276-020-0403-3. 32203102 PMC7156741

