# Supplementary material for: Pollen Emissions of Subpollen Particles and Ice Nucleating Particles
Source: ACS Earth Space Chem. 2023 Apr 27;7(6):1207–18. doi: 10.1021/acsearthspacechem.3c00014 (PMC10863449; doi:10.1021/acsearthspacechem.3c00014)
Supplement: Supplementary file 1 — sp3c00014_si_001.pdf [file sp3c00014_si_001.pdf]

Supplemental Information:

Pollen Emissions of Subpollen Particles and Ice Nucleating Particles

*Brianna H. Matthews[1**], Alyssa N. Alsante[2], and Sarah D. Brooks[1*]*

[1]Texas A&M University, Department of Atmospheric Sciences, College Station, TX, 77843, USA

[2]Texas A&M University, Department of Oceanography, College Station, TX, 77843, USA

* Correspondence to: Sarah D. Brooks – sbrooks@tamu.edu

**Now at Savannah River National Laboratory, Aiken, SC, 29808

Table S1. Meteorological parameters measured during ryegrass sample collection (Easterwood airport measurement, Kestrel Weather Meter measurement).

Date	5/19/21	5/20/21	5/22/21	5/23/21	5/24/21	5/25/21
Time	9:19 am	10:06 am	10:35 am	9:45 am	10:24 am	9:33 am
Relative Humidity	87%, 76%	80%, 75%	76%, 80%	92%, 88%	89%, 80.6%	88%, 79.8%
Temperature	20.0 °C, 23.3 °C	22.8 °C, 24.6 °C	26.1 °C, 25.8 °C	21.7 °C, 22.6 °C	24.4 °C, 24.6 °C	25.0 °C, 26.2 °C
Wind Speed	2.2 m/s, N/A	0.4 m/s, N/A	6.7 m/s, N/A	1.8 m/s, N/A	4.5 m/s, N/A	4.5 m/s, N/A
Wind Direction	SE, N/A	W, N/A	E, N/A	ENE, N/A	ESE, N/A	SSE, N/A
Dew Point Temperature	17.8 °C, 19.1 °C	18.9 °C, 19.7 °C	21.7 °C, 22.8 °C	20.6 °C, 20.6 °C	22.2 °C, 21.4 °C	22.8 °C, 22.9 °C
Wet Bulb Temperature	N/A, 20.6 °C	N/A, 21.3 °C	N/A, 23.9 °C	N/A, 21.2 °C	N/A, 22.5 °C	N/A, 24.0 °C
UV Index	2, N/A	2, N/A	2, N/A	1, N/A	2, N/A	1, N/A
Pressure	101,185.3 Pa, 99,763.0 Pa	101,422.3 Pa, 100,135.5 Pa	101,998.0 Pa, 100,711.2 Pa	102,099.6 Pa, 100,880.5 Pa	101,896.4 Pa, 100,609.6 Pa	101,828.7 Pa, 100,541.9 Pa
Precipitation	none	none	none	light rain	none	none
Sun	up	up	up	up	up	up
Additional Notes	storm previous night	N/A	sprinkled that morning	N/A	N/A	N/A

Table S2. Meteorological parameters measured during ragweed sample collection (Easterwood airport measurement, Kestrel Weather Meter measurement).

Date	9/13/21	9/15/21	9/17/21	9/20/21	9/21/21	9/22/21	9/24/21
Time	10:51 am	12:58 pm	12:01 pm	1:18 pm	12:25 pm	12:49 pm	12:19 pm
Relative Humidity	76%, 74.3%	69%, 72.2%	60%, 72.3%	47%, 71%	54%, 75%	25%, 47.5%	38%, 46%
Temperature	26.1 °C, 27.7 °C	25.6 °C, 26.2 °C	29.4 °C, 28.1 °C	35.0 °C, 30.0 °C	32.8 °C, 33.3 °C	27.8 °C, 27.8 °C	27.8 °C, 30.6 °C
Wind Speed	4.9 m/s, N/A	4.5 m/s, N/A	3.1 m/s, N/A	3.6 m/s, N/A	2.7 m/s, N/A	6.3 m/s, N/A	2.2 m/s, N/A
Wind Direction	E, N/A	N, N/A	N, N/A	SSE, N/A	WSW, N/A	N, N/A	SSE, N/A
Dew Point Temperature	21.7 °C, 22.9 °C	19.4 °C, 21.1 °C	21.1 °C, 22.8 °C	22.2 °C, 24.4 °C	22.2 °C, 28.9 °C	6.1 °C, 15.9 °C	11.7 °C, 18.8 °C
Wet Bulb Temperature	N/A, 24.3 °C	N/A, 22.7 °C	N/A, 24.3 °C	N/A, 26.1 °C	N/A, 30.2 °C	N/A, 20.0 °C	N/A, 22.9 °C
UV Index	2, N/A	4, N/A	7, N/A	9, N/A	8, N/A	9, N/A	8, N/A
Pressure	101,794.8 Pa, 100,745.1 Pa	101,083.7 Pa, 100,000.1 Pa	101,557.8 Pa, 100,406.4 Pa	101,117.6 Pa, 100,033.9 Pa	101,727.1 Pa, 100,575.7 Pa	102,336.7 Pa, 101,219.2 Pa	101,930.3 Pa, 100,745.1 Pa
Precipitation	none	none	none	none	none	none	none
Sun	up	up	up	up	up	up	up
Additional Notes	N/A	N/A	N/A	N/A	N/A	N/A	N/A

Table S2 (continued). Meteorological parameters measured during ragweed sample collection (Easterwood airport measurement, Kestrel Weather Meter measurement).

Date	9/27/21	9/28/21	9/29/21	9/30/21	10/4/21	10/5/21
Time	12:45 pm	1:01 pm	1:18 pm	1:04 pm	1:06 pm	12:24 pm
Relative Humidity	46%, 59%	49%, 69%	65%, 75%	65%, 76%	35%, 59%	28%, 50%
Temperature	30.0 °C, 29.0 °C	33.3 °C, 29.4 °C	27.2 °C, 28.1 °C	30.0 °C, 27.8 °C	30.6 °C, 29.4 °C	29.4 °C, 28.3 °C
Wind Speed	2.7 m/s, N/A	2.2 m/s, N/A	1.3 m/s, N/A	calm, N/A	6.7 m/s, N/A	4.5 m/s, N/A
Wind Direction	S, N/A	SE, N/A	WNW, N/A	calm, N/A	NNW, N/A	N, N/A
Dew Point Temperature	17.2 °C, 20.9 °C	21.1 °C, 23.6 °C	20.0 °C, 23.6 °C	22.8 °C, 23.6 °C	13.3 °C, 21.2 °C	8.9 °C, 17.4 °C
Wet Bulb Temperature	N/A, 23.6 °C	N/A, 25.2 °C	N/A, 24.9 °C	N/A, 24.9 °C	N/A, 23.8 °C	N/A, 21.1 °C
UV Index	8, N/A	8, N/A	8, N/A	5, N/A	8, N/A	8, N/A
Pressure	101,592.0 Pa, 100,440.3 Pa	101,185.3 Pa, 100,000.1 Pa	101,151.4 Pa, 100,000.1 Pa	101,422.3 Pa, 100,237.1 Pa	101,693.3 Pa, 100,440.3 Pa	101,693.3 Pa, 100,541.9 Pa
Precipitation	none	none	none	none	none	none
Sun	up	up	up	up	up	up
Additional Notes	N/A	N/A	N/A	N/A	N/A	N/A

Figure S3. SEM photograph of PIXE cascade impactor filter.

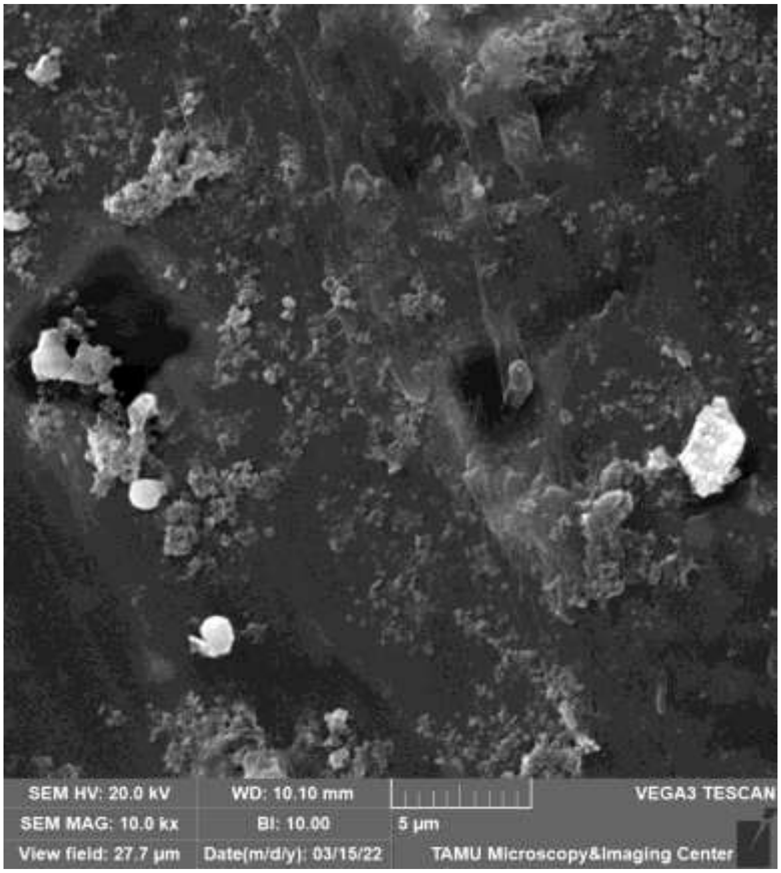

Table S4. Ice Nucleation Experimental Data

Species	Sample Type	Ice Nucleation Mechanism	Mean Nucleation Temperature, ºC	Number of Experiments	Number of Freezing Events
Live Oak	SPPs	Immersion	-29.8 ± 1.7	4	94
Live Oak	Pollen Grains	Immersion	-25.0 ± 1.6	3	74
Live Oak	Pollen Grains	Contact	-22.7 ± 2.2	3	75
Ryegrass	SPPs	Immersion	-30.8 ± 1.0	3	75
Ryegrass	Pollen Grains	Immersion	-28.3 ± 1.9	3	71
Ryegrass	Pollen Grains	Contact	-23.7 ± 0.4	3	75
Giant Ragweed	SPPs	Immersion	-31.3 ± 1.4	4	100
Giant Ragweed	Pollen Grains	Immersion	-27.0 ± 1.3	3	75
Giant Ragweed	Pollen Grains	Contact	-24.6 ± 1.1	3	75

Table S5. Ryegrass and giant ragweed wind-driven SPP emission factors and rates

Species	Relative Humidity	Number of Reproductive Units (counted)	Number of SPPs per Sample (calculated)	Number of SPPs per Pollen Grain (calculated)	Number of SPPs per m^2 (calculated)	1-hour SPP Mean (calculated)	1-hour SPP Peak (calculated)
Ryegrass*	>95%	121	$3.4x10^{12} \pm 1.7x10^{12}$	$7.6x10^{3} \pm 3.8x10^{3}$	$7.4x10^{13} \pm 3.7x10^{13}$	$2.8x10^{11}$	$1.2x10^{11} \pm 2.4x10^{10}$
Ryegrass	>95%	167	$4.7x10^{12}$	$7.7x10^{3}$	$1.0x10^{14}$	$2.6x10^{11}$	$7.2x10^{10} \pm 2.5x10^{10}$
Ryegrass	>95%	168	$2.7x10^{12}$	$4.3x10^{3}$	$5.8x10^{13}$	$1.5x10^{11}$	$4.2x10^{10} \pm 6.7x10^{9}$
Ryegrass	>95%	196	$2.7x10^{12}$	$3.7x10^{3}$	$5.8x10^{13}$	$1.5x10^{11}$	$3.7x10^{10} \pm 1.3x10^{10}$
Ryegrass	>95%	138	$2.1x10^{12}$	$4.2x10^{3}$	$4.6x10^{13}$	$1.2 x10^{11}$	$2.8x10^{10} \pm 5.4x10^{9}$
Giant Ragweed	>95%	113	$1.2x10^{12}$	$4.6x10^{4} – 2.0x10^{5}$	$9.7x10^{14}$	$6.9x10^{10}$	$1.7x10^{10} \pm 6.8x10^{9}$
Giant Ragweed	>95%	107	$1.6x10^{12}$	$6.2x10^{4} – 2.7x10^{5}$	$8.6x10^{14}$	$8.8x10^{10}$	$2.1x10^{10} \pm 4.5x10^{9}$
Giant Ragweed**	>95%	155	$2.6x10^{12} \pm 4.8x10^{9}$	$6.9x10^{4} – 3.0x10^{5}$	$8.8x10^{14} \pm 1.7x10^{12}$	$1.4x10^{11}$	$3.8x10^{10} \pm 6.7x10^{9}$
Giant Ragweed	>95%	109	$2.9 x 10^{12}$	$1.1x10^{5} – 4.8x10^{5}$	$1.2x10^{15}$	$1.6x10^{11}$	$4.5x10^{10} \pm 1.7x10^{10}$
Giant Ragweed	>95%	263	$3.4 x 10^{12}$	$5.3x10^{4} – 2.3x10^{5}$	$6.0x10^{14}$	$1.9x10^{11}$	$4.7x10^{10} \pm 9.9x10^{9}$
Giant Ragweed	>95%	141	$2.1 x 10^{12}$	$6.3x10^{4} – 2.7x10^{5}$	$1.8x10^{15}$	$1.2x10^{11}$	$3.1x10^{10} \pm 9.7x10^{9}$
Giant Ragweed	>95%	105	$1.4 x 10^{12}$	$5.4x10^{4} – 2.3x10^{5}$	$1.5x10^{14}$	$7.6x10^{10}$	$1.8x10^{10} \pm 9.5x10^{9}$
Giant Ragweed	<80%	132	$5.5 x 10^{12}$	$1.7x10^{5} – 7.4x10^{5}$	$2.5x10^{15}$	$3.0x10^{11}$	$7.9x10^{10} \pm 2.0x10^{10}$
Giant Ragweed	<80%	159	$3.5 x 10^{12}$	$9.1x10^{4} – 3.9x10^{5}$	$5.2x10^{14}$	$1.9x10^{11}$	$4.9x10^{10} \pm 2.0x10^{9}$

Giant Ragweed	<80%	187	5.4×10^{12}	$1.2 \times 10^{5} - 5.2 \times 10^{5}$	3.9×10^{15}	3.0×10^{11}	$7.4 \times 10^{10} \pm 4.0 \times 10^{9}$

* Experiment had a tank malfunction which shortened the experiment time by 6 hours, an error is included on the values of this experiment for an adjustment to match 18 hours of data in other experiments

** Experiment had a tank malfunction which shortened the experiment time by 2 minutes, an error is included on the values of this experiment for an adjustment to match 18 hours of data in other experiments
